# Notes and Notices

**Published:** 1892-01

**Authors:** 


### NOTES AND NOTICES.

Dr. Chas. M. Thomas, of 1623 Arch Street, Philadelphia, desires to announce to his professional friends, that he will in future relinquish his general surgical practice, and devote his entire attention to Diseases of the Eye and Ear.

This course he has felt himself obliged to take, as he finds it impossible to conduct both branches of practice, and do full justice to his patients and himself.

December, 1891.

Dr. Horace P. Holmes has removed from Douglas Block to Rooms 201, 202, 203 of Boyd's New Theatre Building, Omaha, Nebraska.

Dr. George H. Clark, formerly associate editor of this journal, has removed from West Walnut Lane and Green Street to No. 4 West Walnut Lane, Germantown, Philadelphia.

